# Research on a novel and improved incremental conductance method

**DOI:** 10.1038/s41598-022-20133-7

**Published:** 2022-09-20

**Authors:** Chunhu Sun, Jing Ling, Jing Wang

**Affiliations:** School of Electronic Engineering, Chaohu University, Chaohu, Anhui China

**Keywords:** Energy harvesting, Renewable energy

## Abstract

Aiming at the problem that the steady-state error and response speed cannot be taken into account in the traditional fixed-step incremental conductance method, combined with the current–voltage (I–U) characteristic curve of photovoltaic (PV) panel, this paper proposes a novel and improved variable-step incremental conductance method. The proposed tracking strategy of maximum power point (MPP) analyzes the I–U characteristic curve, and divides the I–U characteristic curve into four sections, each section has a different variable step size, thus realizing the variable step size control of global four-section. The traditional incremental conductance method and the improved incremental conductance method are simulated under the environment of Matlab/Simulink. The simulation results show that when the light intensity changes rapidly, the novel improved incremental conductance method not only has the advantages of no oscillation in steady state and fast response speed, but also improve the efficiency of photovoltaic power generation, which better realizes the fast and accurate tracking of MPP.

## Introduction

In order to improve the output power of photovoltaic power generation systems, in recent years, many domestic and foreign scholars have conducted in-depth research on the maximum power point tracking (MPPT) algorithm. From the traditional MPPT algorithm, such as constant voltage method, perturb and observation method (P&O), incremental conductance method (INC), to the later improved MPPT algorithm, such as variable step size MPPT control method, fuzzy logic control method, neural network control method, etc. Today, the improved MPPT algorithm has made new progress, which mainly focuses on Multi-Peak MPPT Control Algorithm and Single-Peak MPPT Control Algorithm.

The research situation of Multi-peak MPPT control strategy is as follows. Reference^1^ proposes a hybrid control algorithm that combines the tree species algorithm with the disturbance observation method, which can well realize the tracking control of the maximum power point under the condition of photovoltaic multi-peak output, and has the advantages of fast, accurate and low oscillation. A novel hybrid "Cauchy and Gaussian sine cosine optimization" (CGSCO) algorithm is proposed for MPPT in reference^2^, which show less complexity and computational burden, and has better simulation effect in shaded and unshaded conditions. In reference^3^, a particle swarm optimization algorithm based on incremental conductance was proposed, which can effectively track the global MPP from the multi-peak output power curve and avoid falling into local optimum.

The research situation of Single-peak MPPT control strategy is as follows. Reference^4^ summarizes the research status of MPPT algorithms at home and abroad, and it discusses and compares the advantages and disadvantages of common photovoltaic power generation MPPT algorithms under uniform illumination conditions, the development trend of MPPT algorithms is also mentioned. Reference^5^ proposes an interleaved parallel boost circuit as the front-end circuit, which shortens the tracking time and improves the system efficiency. The improved variable-step incremental conductance method has a faster tracking speed and better stability. Reference^6^ uses the incremental conductance method for secondary search, which can not only accurately find the global optimal solution, but also improve the efficiency of MPPT control.

Although these traditional and improved MPPT algorithms can well achieve maximum power point (MPP) tracking, under the conditions of rapid changes in light intensity. these conventional MPPT algorithms will temporarily fail, and produce oscillations, which makes it impossible to Accurate tracking of MPP. For this reason, many scholars have also carried out research and exploration on this situation. References^7,8^ establish a variable weather parameter (VWP) interval with the control quantity corresponding to the ideal MPP as the center, and limit the MPP optimization range within this interval, which improves the rapidity of system tracking and the accuracy of the system tracking. References^9,10^ build a mathematical model between the perturbation step size of the MPPT algorithm and the light irradiation intensity, ambient temperature, maximum power point and sampling period based on the basic circuit equation of the solar cell. The perturbation step size mathematical model can effectively reduce oscillation phenomenon, which has high reliability and practicability. References^11,12^ studied the maximum power tracking method under different weather, illumination and temperature conditions, which effectively improved the stability of the system. References^13,14^ improved the traditional power disturbance-perturb and observation method (dP-P&O) algorithm and proposed power disturbance-perturb and observation method-power disturbance (dP-P&O-dP) maximum power tracking algorithm. The variable-step tracking method takes into account the fastness and accuracy of tracking. Reference^15^ proposed that when the operating point is located on the left side of the MPP, the step size coefficient is designed as a logarithmic function value, and when the operating point is located on the right side of the MPP, the exponential function value is used as the step size coefficient, which well satisfies the photovoltaic P–U curve, effectively reduces the steady-state oscillation. Reference^16^ proposes to combine the constant pressure method with the double exponential function variable step method, which improves the tracking speed and accuracy. Reference^17^ deduces a variable-step voltage equation based on the incremental conductance method through solar cell parameter modeling, and then solves the output voltage U_ref_. It uses U_ref_ as the starting voltage, to perform MPPT by using the incremental conductance method, which can track MPP quickly and accurately. The step size proposed in reference^18^ is not only related to dP, but also to the difference between dU and dI, and the step size coefficient is variable, which further improves the photovoltaic power generation efficiency. Reference^19^ introduced a variable-step fractional-order incremental conductance method for maximum power point tracking to find the optimal fractional integrator order and fractional derivative order by using a fractional-order PID controller tuned by biomimetic particle swarm optimization. The method show good steady-state, dynamic performance and fast convergence. Reference^20^ proposed an improved three-stage MPPT control strategy. In the non-MPP section, the constant voltage method is used to speed up the tracking speed; in the MPP-like section, the incremental conductance method with variable step size is used to reduce the tracking time; the MPP section adopts Particle swarm optimization algorithm to improve tracking accuracy.

On the basis of referring to the above references, for traditional incremental conductance method and PV panel I–U characteristics, the proposes MPPT divides the I–U curve in four intervals and considers different step sizes for each of them. Simulations were carried out for verifying the feasibility of the method. The novel improved incremental conductance method not only realizes the fast and accurate tracking of MPP, has the advantages of no oscillation in steady state and fast response speed, but also improve the efficiency of photovoltaic power generation.

## Introduction of photovoltaic power generation system

**Figure 1:**
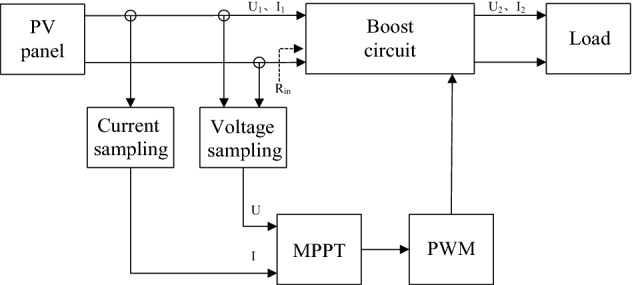
Composition of photovoltaic power generation system.

The photovoltaic power generation system usually consists of PV panel, Boost circuit, MPPT circuit, PWM drive and load, as shown in Fig. 1. The PV panel absorbs solar energy to generate electricity and outputs DC power; the Boost circuit is used to increase the output voltage of the PV panel; the MPPT circuit is used to realize that the photovoltaic power generation system can always output the maximum power; the PWM drive is used to convert the control signal obtained by the MPPT algorithm into PWM signal drives the Boost circuit.

### Principle of maximum power output of PV panel

According to Fig. 1, define R_in_ as the input resistance, and have:

1 $$ R_{{{\text{in}}}} = \frac{{U_{1} }}{{I_{1} }} $$

Assuming that the power transmission of the boost circuit is lossless, then according to the power balance relationship, we can get:

2 $$ U_{1} \times I_{1} = U_{2} \times I_{2} $$

The voltage input and output relationship of the Boost circuit (D represents the duty cycle) is as follows:

3 $$ U_{2} = \frac{{U_{1} }}{1 - D} $$

Assuming that the load is a fixed resistance R, then according to Fig. 1, it can be known that:

4 $$ U_{2} = I_{2} \times R $$

Combining formulas (1) to (4), the input resistance R_in_ can be obtained as:

5 $$ R_{{{\text{in}}}} = R \times (1 - D)^{2} $$

**Figure 2:**
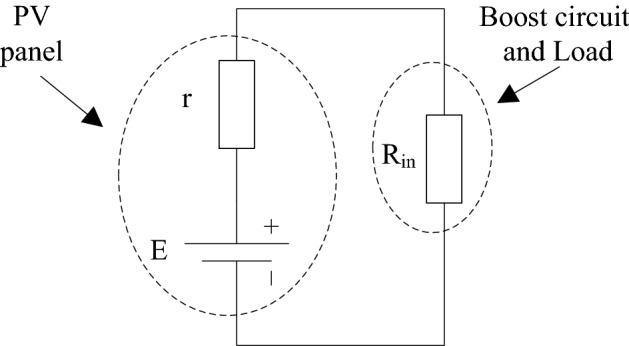
Equivalent diagram of the main circuit of the photovoltaic power generation system.

From the conclusion of formula (5), the Boost circuit and the load in the main circuit in Fig. 1 can be equivalent to the input resistance R_in_, and the PV battery is equivalent to the DC power supply E and the internal resistance r. The resulting equivalent main circuit is shown in Fig. 2.

From the analysis of Fig. 2, it can be concluded that when the internal resistance r and the input resistance R_in_ are equal, the output power of the PV panel is the largest, at this time:

6 $$ {\text{r}} = R \times (1 - D)^{2} $$

According to formula (6), the duty cycle D can be solved as:

7 $$ D = 1 - \sqrt {\frac{{\text{r}}}{R}} $$

Since the resistance R is fixed, the internal resistance r is a dynamic resistance, which is constantly changing, and its size is determined by the light intensity and temperature. It can be seen from formula (7) that under different lighting and temperature conditions, the internal resistance r and the duty cycle D are in one-to-one correspondence. As long as the duty cycle D is controlled according to formula (7), the PV battery can output the maximum power.

### PV panel output characteristics

The output characteristics of PV panel include P–U output characteristics and I–U output characteristics, both of which are affected by light intensity and temperature. The following analysis is made from the two aspects of light intensity and temperature. When the light intensity changes and when the temperature changes, the influence of P–U output characteristics and I–U output characteristics on PV panel.

**Figure 3:**
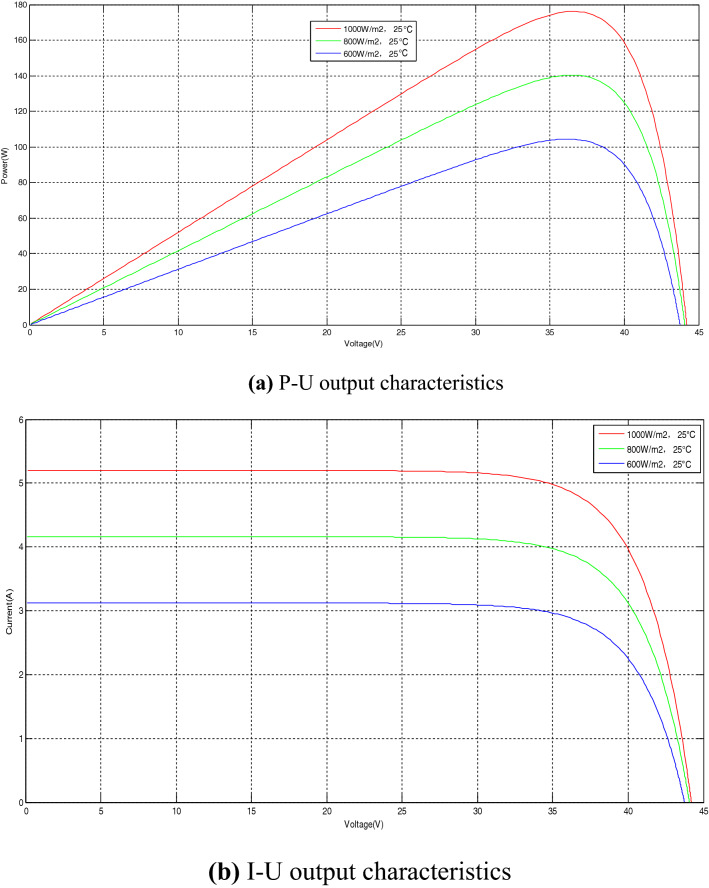
The output characteristics of PV panel when the light intensity changes.

When the temperature is constant at 25 °C and the light intensity is 1000 W/m^2^, 800 W/m^2^, and 600 W/m^2^, respectively, the P–U output characteristics and I–U output characteristics of PV panel are shown in Fig. 3.

It can be seen from Fig. 3 that when the temperature is constant and the light intensity gradually increases, the P–U output characteristic curve and the I–U output characteristic curve of the PV panel both move up significantly, and the output power of the PV panel increases.

**Figure 4:**
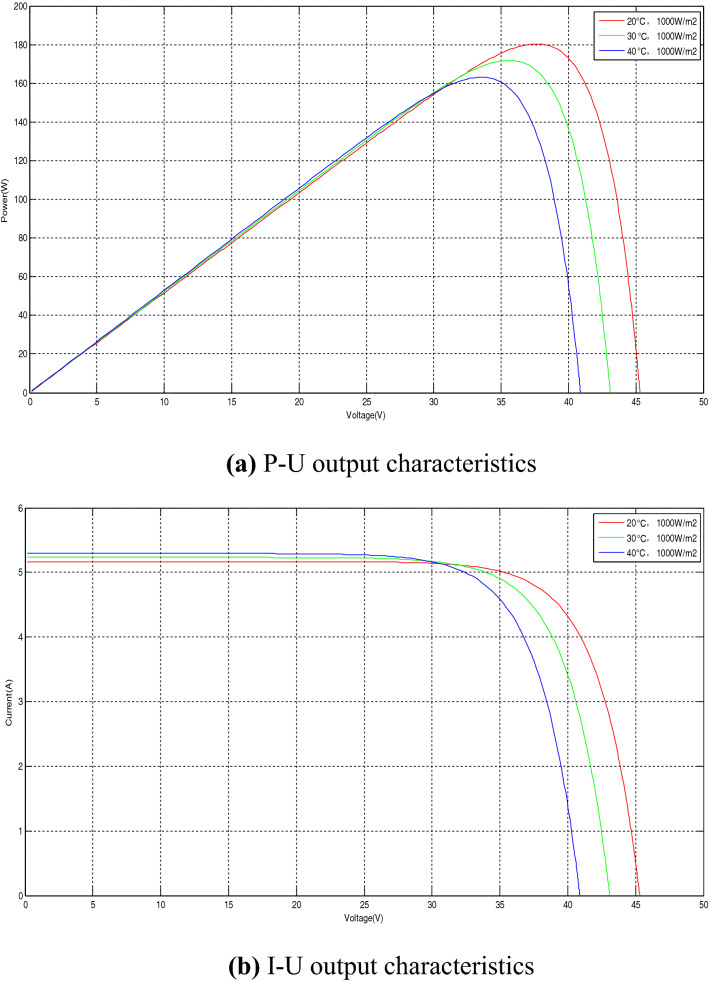
The output characteristics of PV panel when the temperature changes.

When the light intensity is constant at 1000 W/m^2^ and the light intensity is 20 °C, 30 °C, and 40 °C, respectively, the P–U output characteristics and I–U output characteristics of PV panel are shown in Fig. 4.

It can be seen from Fig. 4 that when the light intensity is constant and the temperature gradually increases, the P–U output characteristic curve and the I–U output characteristic curve of the PV panel both shift significantly to the left, and the output power of the PV panel decreases.

## Traditional incremental conductance method

It can be seen from the P–U output characteristic curve of the PV panel that when the operating point is located to the left of the maximum power point, as the voltage increases, the slope value of the curve (dP/dU) is greater than 0 and gradually becomes smaller; when the operating point is located at the maximum power point, the slope value of the curve (dP/dU) is equal to 0; when the operating point is located to the right of the maximum power point, as the voltage increases, the slope value of the curve (dP/dU) is less than 0 and gradually becomes smaller.

For the slope value of the curve (dP/dU), there is the following mathematical relationship:

8 $$ \frac{dP}{{dU}} = \frac{d(UI)}{{dU}} = U\frac{dI}{{dU}} + I $$

When the operating point is at the maximum power point, the slope value of the curve (dP/dU) is 0, then there are:

9 $$ U\frac{dI}{{dU}} + I = 0 $$

Divide formula (9) by the voltage U, we can get:

10 $$ \frac{dI}{{dU}} + \frac{I}{U} = 0 $$

The position of the current operating point relative to the maximum power point can be determined by formula (10):

$$ \frac{dI}{{dU}} + \frac{I}{U} > 0\;\;\;\;\;\;\;{\text{Left}}\;{\text{side}}\;{\text{of}}\;{\text{maximum}}\;{\text{power}}\;{\text{point}} $$

11 $$ \frac{dI}{{dU}} + \frac{I}{U} = 0\;\;\;\;\;{\text{maximum}}\;{\text{power}}\;{\text{point}} $$

$$ \frac{dI}{{dU}} + \frac{I}{U} < 0\;\;\;\;\;\;\;{\text{Right}}\;{\text{side}}\;{\text{of}}\;{\text{maximum}}\;{\text{power}}\;{\text{point}} $$

**Figure 5:**
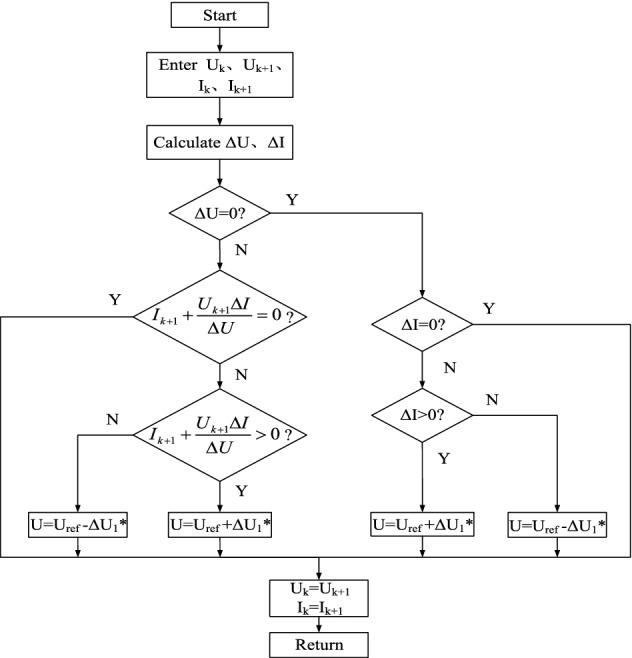
Flow chart of traditional incremental conductance method.

The control flow chart of the traditional conductance increment method is shown in Fig. 5.

In Fig. 5, ΔU_1_* is a fixed step size, and ΔU and ΔI are respectively expressed as:

12 $$ \Delta U = U_{k + 1} - U_{k} $$

13 $$ \Delta I = I_{k + 1} - I_{k} $$

After each disturbance, the U(k) and I(k) values need to be updated. Although the traditional incremental conductance method is simple to control, when the step size is too large, the steady-state oscillation is large; if the step size is too small, the tracking speed is too slow, and the oscillation phenomenon cannot be eliminated.

## Improved incremental conductance method

The oscillation phenomenon is caused by the unreasonable control of the perturbation step size. In order to solve the oscillation problem caused by the fixed step size of the traditional incremental conductance method, this paper proposes a novel improved incremental conductance method based on the traditional incremental conductance method, and the interval division of I–U characteristic curve and the design of variable step size were studied.

According to formula (10), the function f(U, I) is defined as follows:

14 $$ f(U,I) = \frac{dI}{{dU}} + \frac{I}{U} $$

**Figure 6:**
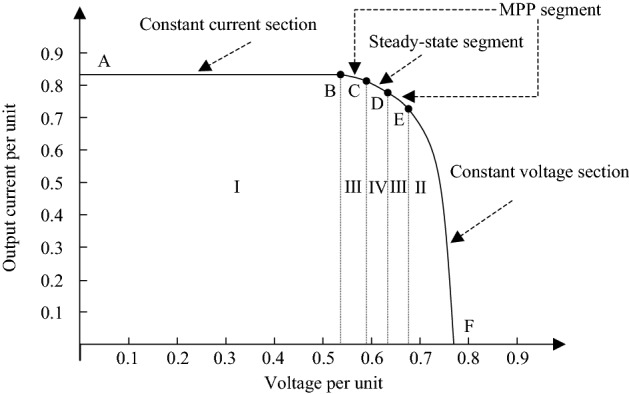
I–U characteristic curve interval division diagram.

Next, the I–U characteristic curve of the photovoltaic battery is divided into sections, and the section division diagram is shown in Fig. 6.

As shown in Fig. 6, the I–U characteristic curve is divided into four sections, the AB section is approximately the constant current section, the EF section is approximately the constant voltage section, the BC and DE sections are the MPP section, and the CD section is the steady state section. Next, the function f(U, I) value of the four-segment interval is analyzed, and the variable step size is designed at the same time.

### Analysis of constant current section

The current operating point is on the left side of the maximum power point. Since the I–U curve of photovoltaic battery has constant current characteristics, the current I value is approximately unchanged, and the dI value is about 0. Then the value of the function f(U, I) can be approximated as:

15 $$ f(U,I) \approx \frac{I}{U} > 0 $$

Combined with formula (15), when the operating point moves to the right, it can be known from the I–U curve characteristics of the photovoltaic battery: the current I value remains unchanged, the voltage U value gradually increases, so the f(U, I) value gradually decreases, if the value of the f(U, I) satisfies:

16 $$ f(U,I) > 0.5 $$

At this time, it can be considered that the current operating point is in the constant current stage, and the variable step size γ_1_ of the constant current stage can be designed as:

17 $$ \gamma_{1} = \alpha_{1} \times \frac{I}{U} $$

In the formula, α_1_ represents the step size factor, which is a small positive number.

### Analysis of constant voltage section

The current operating point is in the region to the right of the maximum power point. Since the I–U curve of the photovoltaic cell has constant voltage characteristics, the voltage U value is approximately unchanged, and the dU value is about 0. Then the dI/dU value will be negative infinity, and the I/U value is The fixed value can be ignored, and the value of the function f(U, I) can be approximated as:

18 $$ f(U,I) \approx \frac{dI}{{dU}} < 0 $$

Combined with formula (18), when the operating point moves to the left, it can be known from the I–U curve characteristics of the photovoltaic battery that the value of voltage dU remains unchanged, while the value of current dI gradually decreases, so the value of the f(U, I) gradually increases. The value of the f(U, I) satisfies:

19 $$ f(U,I) < - 0.5 $$

At this time, it can be considered that the current operating point is in the constant pressure section, and the variable step size γ_2_ in the constant pressure section can be designed as:

20 $$ \gamma_{2} = \alpha_{2} \times \left| {\frac{dI}{{dU}}} \right| $$

In the formula, α_2_ represents the step size factor, which is a small positive number.

### Analysis of MPP segment

The current operating point is in the vicinity of the maximum power point. It can be seen from formula (10) that when the operating point is in the MPP segment, the value of the function f(U, I) tends to 0. If the value of the f(U, I) satisfies:

21 $$ 0.05 < f(U,I) \le 0.5OR{ - }0.5 \le f(U,I) < { - }0.05 $$

At this time, it can be considered that the current working point is in the MPP segment, and the variable step size γ_3_ of the MPP segment can be designed as:

22 $$ \gamma_{3} = \alpha_{3} \times \left| {\frac{dI}{{dU}} + \frac{I}{U}} \right| $$

In the formula, α_3_ represents the step size factor, which is a small positive number. It can be seen from formula (22) that when the operating point is close to the maximum power point, the step size γ_3_ gradually decreases and tends to 0.

### Analysis of steady-state segment

In the steady state, in order to avoid repeated oscillation of the operating point in the steady state, the output duty cycle of the controller should be kept unchanged, and the disturbance should be stopped. The disturbance step size γ_4_ should be taken as:

23 $$ \gamma_{4} = 0 $$

In steady state, the value of the f(U, I) should satisfy:

24 $$ - 0.05 \le f(U,I) \le 0.05 $$

**Figure 7:**
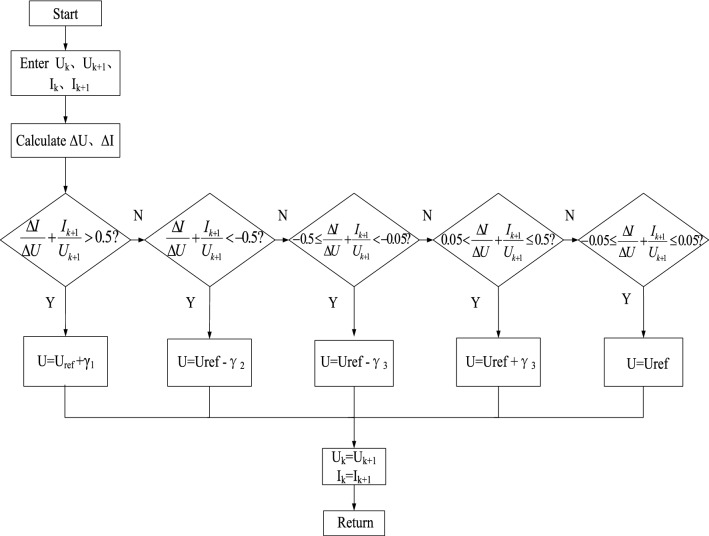
Flow chart of the variable-step conductance increment method.

The control flow chart of the variable-step incremental conductance method is shown in Fig. 7.

The variable-step conductance increment method and the variable-step design not only avoid the oscillation phenomenon in steady state, but also improve the rapidity and accuracy of photovoltaic power generation system tracking.

## Simulation analysis

### P–U characteristics simulation analysis

**Figure 8:**
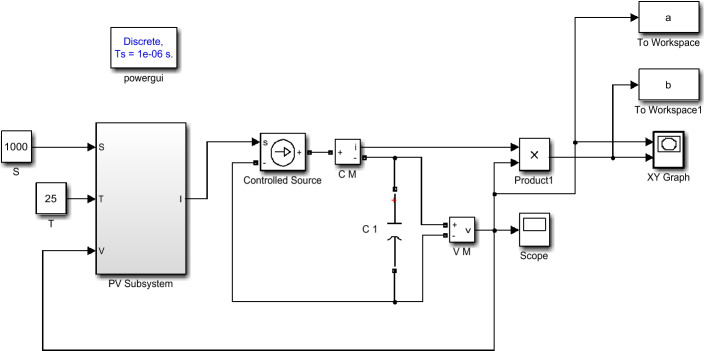
P–U characteristics simulation model.

At a constant temperature of 25 °C, the P–U characteristics of PV panel were simulated and analyzed, when the light intensity was 1000 W/m^2^, 800 W/m^2^, and 600 W/m^2^, respectively. The simulation model is shown in Fig. 8.

As shown in Fig. 8, the output voltage and output power of the PV panel are sent to the workspaces a and b respectively, which are used to draw the P–U characteristic curve of the PV panel. PV panel simulation model parameters are shown in Table 1.

**Table 1:** PV panel simulation model parameters.

I_m_	V_m_	V_oc_	I_sc_	S_ref_	T_ref_
4.96A	35.2 V	44.2 V	5.2 A	1000 W/m^2^	25 °C

**Figure 9:**
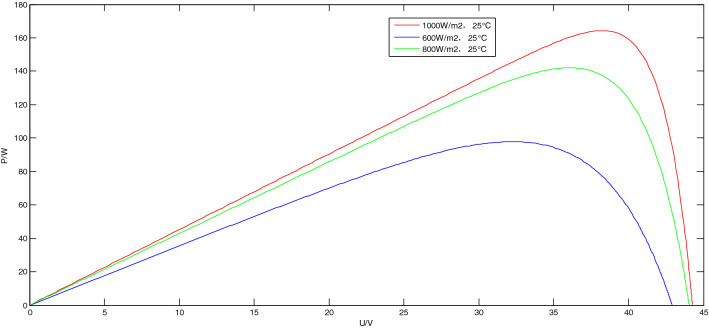
The P–U characteristic curve under different light intensities.

The P–U characteristic curve under different light intensities are shown in Fig. 9.

It can be obtained from the Fig. 9: the power and voltage of the maximum power point under different light intensities, as shown in Table 2.

**Table 2:** The power and voltage of the maximum power point under different light intensities.

Light intensities	1000 W/m^2^	600 W/m^2^	800 W/m^2^
Power	164.2 W	97.8 W	142.1 W
Voltage	38.3 V	32.2 V	36.1 V

### System simulation model

**Figure 10:**
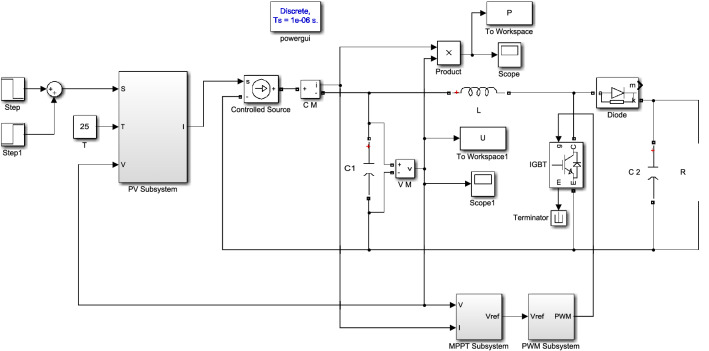
System simulation model.

The system simulation model is shown in Fig. 10, which consists of PV subsystem, Boost circuit, load R, MPPT subsystem and PWM subsystem. The traditional incremental conductance method is a fixed step, the step value ΔU_1_* is 0.01, the variable step incremental conductance method is a variable step, and the step factors α_1_, α_2_, α_3_ are fixed values of 0.05, 0.02, 0.01, respectively. In Fig. 10, L1 = 5e−4H, C_1_ = C_2_ = 5e−4F, R = 10 Ω.

The MPPT subsystem collects voltage and current, and obtains the reference voltage control signal U_ref_, through the MPPT algorithm. The control signal U_ref_ is compared with the sawtooth wave with a frequency of 10 kHz through a comparator. If meets the relationship more than, it outputs a high level, and if it meets the relationship less than, it outputs a low level, thus generating a PWM signal. The PWM signal drives the IGBT to realize pulse width modulation of Boost circuit,Therefore, the Boost circuit can output different voltages.

### System simulation analysis

In order to simulate the MPPT tracking effect of the system under the conditions of rapid illumination changes, Simulink simulation was carried out for the system. During the simulation, the temperature was constant at 25 °C, but the light intensity changed rapidly.

During the simulation, the change rule of the light intensity is as follows: in 0 ~ 0.3 s, the light intensity is constant at 1000 W/m^2^; in 0.3 ~ 0.6 s, the light intensity is constant at 600 W/m^2^; in 0.6 ~ 1 s, the light intensity is constant at 800 W/m^2^; The simulation time is 1 s, the ode45 algorithm is used, and the sampling time is 1e−6 s.

#### Simulation analysis of the output voltage

**Figure 11:**
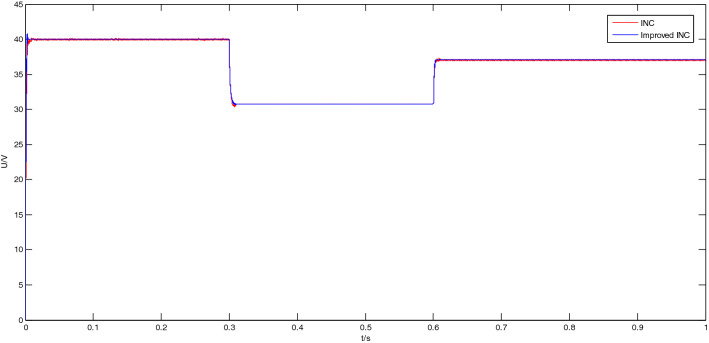
Output voltage simulation results of two conductance increment methods.

The MPPT simulation analysis of the output voltage of the PV panel was carried out by using the traditional incremental conductance method and the improved incremental conductance method respectively. The MPPT simulation results of the two incremental conductance methods are shown in Fig. 11.

It can be seen from Fig. 11 that the output voltage curves of the traditional conductance incremental method and the improved conductance incremental method almost overlap, and the voltage values are almost the same. The output voltage and error are shown in Table 3.

**Table 3:** Output voltage of two conductance increment methods under different light intensities.

Light intensity	1000 W/m^2^	600 W/m^2^	800 W/m^2^
Voltage	40.0 V	30.7 V	37.1 V
Error	4.44%	4.66%	2.77%

From Table 3, it can be concluded that under different lighting conditions, the maximum power point voltage tracking error of the conductance incremental method and the improved conductance incremental method are both within the error range of 4.66%, the error is small, and the voltage tracking effect is good and stable.

#### Simulation analysis of the output power

**Figure 12:**
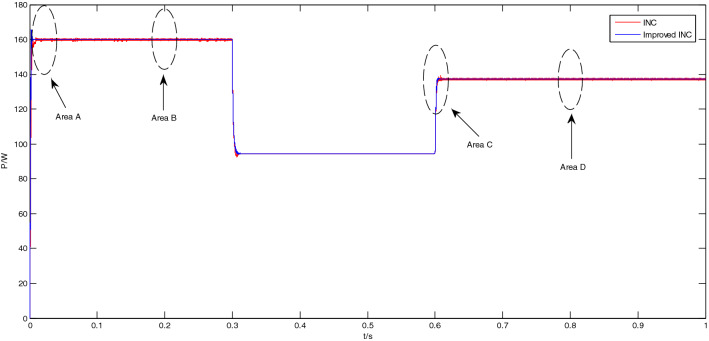
MPPT simulation results of two conductance increment methods.

The MPPT simulation analysis of the output power of the PV panel was carried out by using the traditional incremental conductance method and the improved incremental conductance method respectively. The MPPT simulation results of the two incremental conductance methods are shown in Fig. 12.

It can be seen from Fig. 12 that the output power curves of the traditional conductance incremental method and the improved conductance incremental method almost overlap, and the voltage values are almost the same. The output power and error are shown in Table 4.

**Table 4:** Output power of two conductance increment methods under different light intensities.

Light intensity	1000 W/m^2^	600 W/m^2^	800 W/m^2^
Power	159.9 W	94.4 W	137.4 W
Error	2.62%	3.48%	3.31%

From Table 4, it can be concluded that under different lighting conditions, the power tracking errors of the conductance incremental method and the improved conductance incremental method are both within the error range of 3.48%, the error is small, and the power tracking effect is good and stable.

**Figure 13:**
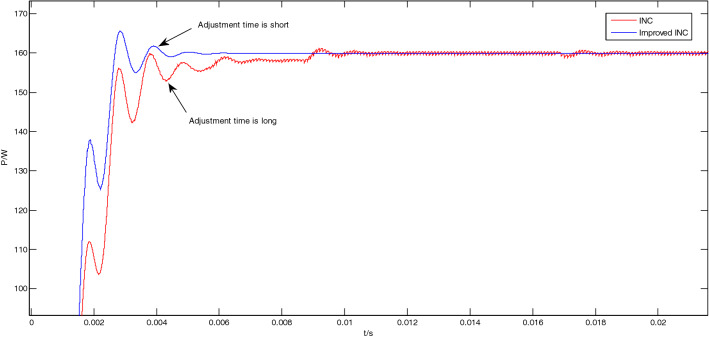
Enlarged view of Area A.

**Figure 14:**
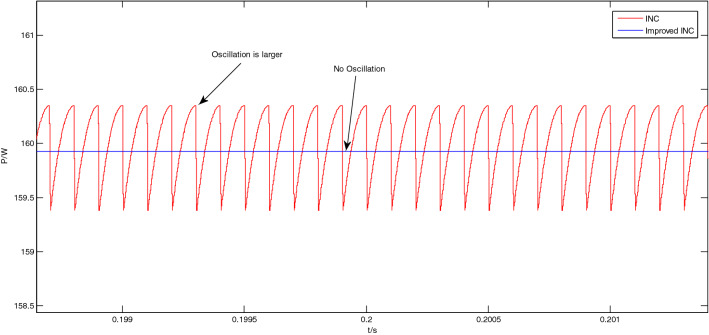
Enlarged view of Area B.

**Figure 15:**
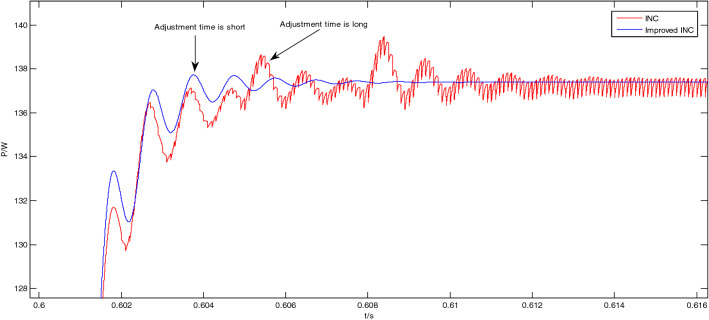
Enlarged view of Area C.

**Figure 16:**
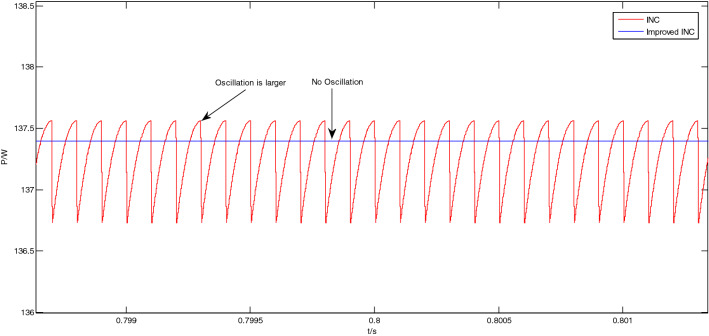
Enlarged view of Area D.

The enlarged views of the Area A to Area D in Fig. 12 are shown in Figs. 13, 14, 15 and 16, respectively.

As shown in Fig. 13, when the light intensity is 1000 W/m^2^, the traditional incremental conductance method enters a steady state at the 0.01 s; However, the improved incremental conductance method has entered a steady state at the 0.005 s, adjustment time is short, and the response speed is fast.

As shown in Fig. 14, at this time, the light intensity is 1000 W/m^2^, and the system is already in a steady state at about 0.2 s. In the steady state, the output power of the traditional incremental conductance method oscillates greatly, and the peak-to-peak value is about 0.96 W; The output power of the improved incremental conductance method also has no oscillation in steady state.

As shown in Fig. 15, when the light intensity increases from 600 to 800 W/m^2^ at 0.6 s, the output power of the the traditional incremental conductance method oscillates greatly, and the system enters the steady state at 0.612 s; while the output power of the improved incremental conductance method oscillates less, the system has entered the steady state at the 0.607 s, adjustment time is short, and the response speed is fast.

As shown in Fig. 16, at this time, the light intensity is 800 W/m^2^, and the system is already in a steady state at about 0.8 s. In the steady state, the output power of the traditional incremental conductance method oscillates greatly, and the peak-to-peak value is about 0.87 W; The output power of the improved incremental conductance method also has no oscillation in steady state.

From Figs. 11, 12, 13, 14, 15 and 16, it can be seen that when the light intensity changes rapidly, the novel and improved incremental conductance method not only has the advantages of no oscillation in steady state and fast response speed, but also the efficiency of photovoltaic power generation is improved, and the fast and accurate tracking of MPP is better achieved.

## Conclusion

On the basis of analyzing the output characteristics of PV panel in photovoltaic power generation systems and the incremental conductance method, this paper proposes a novel improved incremental conductance method with variable step size. The incremental conductance method is simulated by Simulink. The simulation results show that the novel and improved incremental conductance method can not only eliminate the oscillation phenomenon caused by unreasonable step size setting, but also improve the efficiency of the photovoltaic power generation system, and better achieve fast and accurate tracking of the maximum power point, verifies the correctness of the proposed improved incremental conductance method at the same time, which provides a reference for the MPPT control method under the rapid change of light intensity.

## Data Availability

All data generated or analysed during this study are included in this published article.
